# Description of a contemporary pathogenic *Escherichia coli* isolated from pigs with post-weaning diarrhea in the United States from 2010 to 2023

**DOI:** 10.1186/s13567-025-01568-y

**Published:** 2025-07-01

**Authors:** Rodrigo C. Paiva, Eric R. Burrough, Nubia Macedo, Ana Paula S. P. Silva, Maud de Lagarde, John M. Fairbrother, Pablo E. Piñeiro, Marcelo N. Almeida

**Affiliations:** 1 Department of Veterinary Diagnostic and Production Animal Medicine, College of Veterinary Medicine, Iowa State University, Ames, IA USA; 2 OMSA Reference Laboratory for E. Coli, Department of Pathology and Microbiology, Faculty of Veterinary Medicine, Université de Montréal, Saint-Hyacinthe, QC Canada

**Keywords:** *Escherichia coli*, ETEC, STEC, post-weaning colibacillosis, virulence factor

## Abstract

Post-weaning diarrhea (PWD) due to *Escherichia coli* in pigs is a significant enteric disease in the U.S. Contemporary data about the main virulence factors, colony morphology, and distribution of virotypes of isolates associated with post-weaning colibacillosis (PWC) is essential information for swine veterinarians, producers, and stakeholders. This study reports the rate of PWC/PWD, frequency of detection of fimbrial types, pathotypes, toxins, virotypes, morphological characteristics, and temporal analysis of the most prevalent virotype of enterotoxigenic *E. coli* recovered from cases of PWD in pigs in the United States. There was a significant increase in PWC cases submitted to the diagnostic laboratory in 2013, 2014, and 2021, respectively. A greater frequency of detection of F18 fimbrial adhesin (69.87%) was observed compared to F4 (26.19%), F5 (0.25%), F41 (0.13%), AIDA (0.10%), and multiple adhesins (3.46%), respectively. Hybrid ETEC:STEC pathotype was greater than ETEC and STEC, and STb toxin was present in 93.33% of the detected isolates. The most frequently observed virotype was F18:LT:STa:STb:Stx2e (27.71%). Smooth:mucoid colony morphology was associated with a greater likelihood of PWC (0.758, 0.968, and 0.993) compared to smooth, intermediate, and rough, respectively. The frequency of the F18:LT:STa:STb:Stx2e virotype increased across all U.S. states from 2016 to 2023, greatest in the northwest and east of Iowa and northeast Indiana in 2022 and Iowa in 2023. The virulence factors and morphology of enterotoxigenic *E. coli* associated with PWC across different states in the U.S. from 2010 to 2023 were diverse; nevertheless, the virotype F18:LT:STa:STb:Stx2e predominated and increased in frequency during this time period.

**Supplementary Information:**

The online version contains supplementary material available at 10.1186/s13567-025-01568-y.

## Introduction

Post-weaning diarrhea (PWD) associated with pathogenic strains of *Escherichia coli* (*E. coli*) remains one of the most significant enteric diseases in swine production in the United States (U.S.) and worldwide [1–6]. Pathogenic *E. coli* strains have been classified into pathotypes and virotypes based on the production of broad classes of virulence factors and the mechanisms by which they cause disease. In pigs, enterotoxigenic *E. coli* (ETEC), Shiga toxin-producing *E. coli* (STEC), and enteropathogenic *E. coli* (EPEC) are commonly reported pathotypes and are considered the most important [7]. In addition, hybrid pathotypes (e.g., ETEC/STEC) demonstrating a combination of virulence factors associated with diarrheic pigs have been documented in different countries [8–15].

ETEC strains are among the most important in swine [3, 4] and produce enterotoxins (e.g., LT, STa, STb) stimulating fluid secretion, and fimbrial adhesins (e.g., F4 (K88), F18) permitting adherence to the intestinal mucosa, subsequently resulting in diarrhea [2, 4, 16, 17]. F4 (K88) is a common fimbrial adhesin of ETEC associated with high mortality due to diarrhea during lactation and early after weaning, whereas F18 ETEC is more frequently associated with PWD [2, 18]. F18 STEC strains are associated with edema disease (ED) [7]. Other adhesins, such as F5 (K99), F6 (987P), and F41, have been rarely associated with PWD [1, 3, 19–22].

Toxins associated with pathogenic *E. coli* are important for identifying and characterizing pathotypes [23]. F4 (K88) ETEC usually produces both LT and STb enterotoxins, with or without STa, whereas F18 ETEC typically produces STa and STb heat-stable toxins with or without Stx2e toxin [3, 24]. Other toxins and adhesins such as EAST1 and AIDA and porcine attaching and effacing-associated factor (paa) have been found in *E. coli* associated with pigs with diarrhea [6, 7, 25]. However, the role of these virulence factors in the development of porcine diarrhea has not been well characterized [12, 26, 27].

Rapid and accurate confirmatory identification of *E. coli* is now achieved using Matrix Assisted Laser Desorption Ionization-time of flight mass spectrometry (MALDI-TOF MS), generating a specific spectral profile compared with a microorganism database [25, 28]. Nevertheless, the morphology of colonies of pathogenic *E. coli* has been reported to vary from smooth to rough or mucoid [25, 29, 30]. Hemolysis, although not a virulence factor by itself in porcine ETEC and ED strains, has been used as a marker for pathogenicity in isolates producing F4 (K88) and F18 adhesin and certain isolates producing F6 (987P) [25]. Contemporary data on porcine ETEC morphological characteristics and its association with PWC is lacking and may help veterinarians and producers prevent and control this disease.

This study reports the rate of PWC over cases of porcine PWD, the frequency of detection of various fimbrial adhesins, toxins, and virotypes, morphological characteristics of pathogenic *E. coli*, and temporal analysis of the predominant virotype recovered from cases of PWC in pig farms across different U.S. states from 2010 to 2023.

## Materials and methods

### Overview and selection criteria

This study included 3,150 *E. coli* isolates originating from confirmed cases of post-weaning colibacillosis (PWC) from 2010 to 2023 (Additional file 1). Cases originated from different states across the U.S. and were submitted to the Iowa State University Veterinary Diagnostic Laboratory (ISU-VDL) for confirmation.

All cases were associated with a disease diagnostic code (Dx-code) [31], representing confirmation of PWC by a pathologist based on individual case history, histopathological examination, and ancillary laboratory testing, including bacteriological culture and Polymerase Chain Reaction (PCR) [31, 32]. Briefly, the case history was associated with scour and or diarrhea observed clinically and histopathological examination with the observation of short rod-shaped light basophilic bacterial colonies lining the apical brush border and extending to the base of the small intestine villi.

### Isolation and characterization of the isolates

*E. coli* was cultured and isolated following the ISU-VDL standard operating procedure (SOP) and according to methods previously described [33, 34]. Briefly, samples (fecal swabs and fresh intestinal tissue) submitted to ISU-VDL were plated on TSAblood agar plates (5% sheep blood in Tryptic Soy Agar, Remel Inc, San Diego, CA, USA) and incubated at 37 °C for 24 h. Colony morphology, particularly hemolysis (a clear zone around the colony) and classification of smooth:mucoid (slick, moist, with sticky surface with mucoid hemispheres varying from yellow, amber of peach colon), smooth (shiny, glistening, even surface, uniform and flat), intermediate (compact, slightly raised, darker yellow or amber centers and lighter yellow edges), and rough (flat, dry, and spreading, with irregular edges and a sharp, cut-glass appearance, and yellow or amber), were used as a first macroscopic screening [30, 35], and identification of hemolytic *E. coli* isolates was confirmed by matrix-assisted laser desorption ionization time-of-flight mass spectrometry MALDI-TOF MS [28]. All *E. coli* isolates from confirmed clinical cases were submitted to a multiplex PCR to detect fimbrial and toxin genes.

### Characterization of virulence factors by PCR

DNA extracted from the isolates was prepared, and the presence of fimbrial and toxin genes was determined using the ISU-VDL SOP for *Escherichia coli* genotyping by multiplex Gel-based PCR [36]. The fimbrial adhesin and toxin genes included those encoding F4 (K88), F5 (K99), F6 (987P), F18, F41, AIDA, EAEA, PAA, EAST1, LT, STa, STb, Stx1, Stx2, and Stx2e, respectively. The isolated pathotypes and combinations of virulence factors were classified and reported as proposed by Fairbrother and Gyles [7].

### Data management and collation

All cases were selected from the ISU-VDL database between 2010 and 2023. The confirmed PWC cases were sorted out from all porcine PWD cases during the study period. The criteria of PWC included pigs with a clinical history of enteric disease (scours or diarrhea), age of 3 to 8 weeks, histopathological evaluation with confirmation of *E. coli* characteristic intestinal colonization, and ancillary laboratory testing, including bacteriological culture and presence of hemolytic *E. coli* isolates positive for one or more of the tested virulence genes. For each case, one isolate representative of each virotype present was selected. The frequency of detection of fimbriae, toxins, and virotypes was calculated over the study's total isolates (3150). According to the veterinary laboratory data availability, the utilized information regarding colony morphology was from 2021 to 2023.

### Statistical analysis

A Binomial regression model in R was used to estimate the rates of PWC over the total of PWD cases per year, e.g., the PWC by PWD proportion by year was considered the dependent variable, year the independent variable (2010 to 2023) and the PWD as weight variable related to the binomial regression model. SAS.9.4 software [37] was used to calculate the risk ratio of colony morphology associated with confirmed PWC cases over cases of PWD, from 2021 to the end of the study period, 2023, according to laboratory data availability.

A spatial and temporal analysis used the case identifier, year of submission (2020 to 2023), farm addresses, latitude, and longitude data with respect to the isolates possessing the most frequently detected virulence factor combination. Permutation spatial analysis using Ripley’s K function (spatstat version 3.0.6 R package) was used to detect spatial point locations of these isolates in the study.

Duplicated locations were aggregated by case identifiers, the duplicated counts of cases by locations were used as weights in Ripley’s K function. Kernel density estimation (KDE) was used to identify and visualize clustering areas; the density estimation was focused on the Midwest region using likelihood cross-validation to select the bandwidth that maximizes the likelihood of the observed data under the KDE model. The spatial analyses and maps were created using R version 4.3.1 with specific packages “sp,” version 2.0.0, and “sf,” version 1.0.13.

## Results

**Figure 1:**
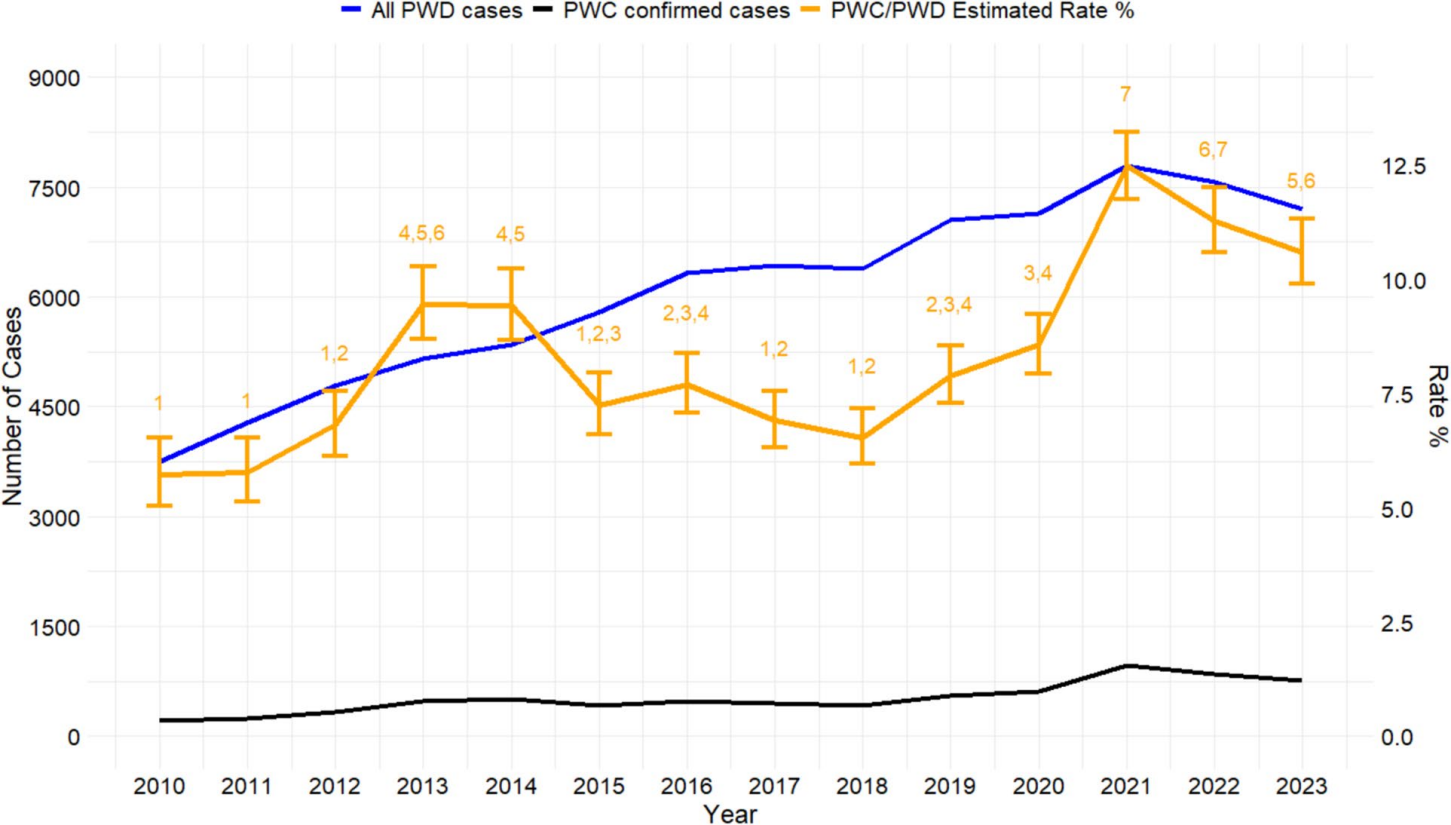
**Porcine enteric cases at the ISU-VDL over the study period.** The solid blue line represents all porcine PWD cases, the solid black line represents the confirmed PWC cases, and the orange line represents the rate of PWC/PWD. The numbers represent the statistical difference between years.

The rate of PWC was numerically higher in 2013 and 2014 and 2021, 2022, and 2023, respectively, with a statistically significant increase in the PWC case rate in 2013, 2014, and 2021 (*P-*value > 0.001), respectively, compared with the previous year (Figure 1).

**Figure 2:**
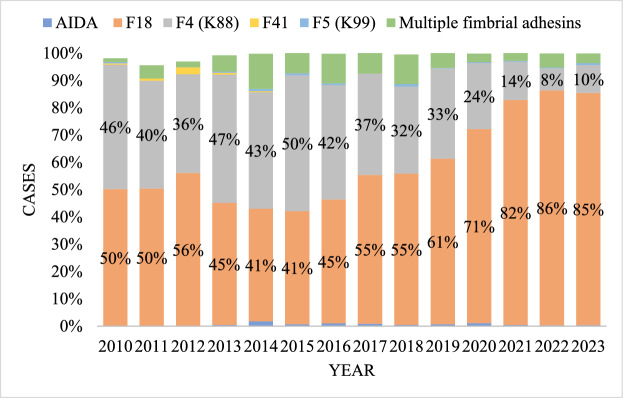
**Pathogenic** ***E. coli* fimbrial type detection over the study period at the ISU-VDL**. Each color represents a different fimbrial type. The percentages represent the main fimbriae types F4 and F18 from PWC cases.

The frequency of detection of F18 fimbria adhesin compared with F4 numerically increased from 2017 to 2022 (Figure 2).

There was great variation in the number of fimbrial types and combinations of pathotypes and toxin genes detected (Table 1). F18 fimbrial adhesin was the most frequently detected (69.87%) compared to all others, and the hybrid ETEC:STEC pathotype was greater (50.13%) compared to ETEC (48.16%), and STEC (1.71%), respectively. A relatively low proportion of virotypes (3.46%) contained multiple fimbrial types (Table 1); STb toxin was present in 93.33% of the overall detection of the isolates (Table 2).

**Table 1:** **Number and frequency of fimbrial type and pathotype detection in pathogenic** ***E. coli*** **isolates from cases of PWC in the U.S. from 2010 to 2023**

Fimbrial adhesin	Pathotype	Number of pathotypes (n)	Frequency of detection (%)
F18	ETEC:STEC	1502	47.68%
	ETEC	645	20.48%
	STEC	54	1.71%
F4 (K88)	ETEC	825	26.19%
F5 (K99)	ETEC	8	0.25%
F41	ETEC	4	0.13%
AIDA	ETEC:STEC	3	0.10%
Multiple adhesins	ETEC	35	1.11%
	ETEC:STEC	74	2.35%

**Table 2:** **Number and frequency of toxins and non-fimbrial adhesins detected in pathogenic** ***E. coli* isolates from cases of PWC in the U.S. from 2010 to 2023.**

Toxins and non-fimbrial adhesins	Number of isolates possessing toxin or non-fimbrial adhesin genes(n)	Frequency of detection (%)
STb	2940	93.33%
LT	2819	89.49%
STa	1994	63.30%
EAST1	1692	53.71%
Stx2e	1581	50.19%
Stx2	1528	48.51%
Paa	674	21.40%
Stx1	525	16.67%
EAEA	11	0.35%

**Figure 3:**
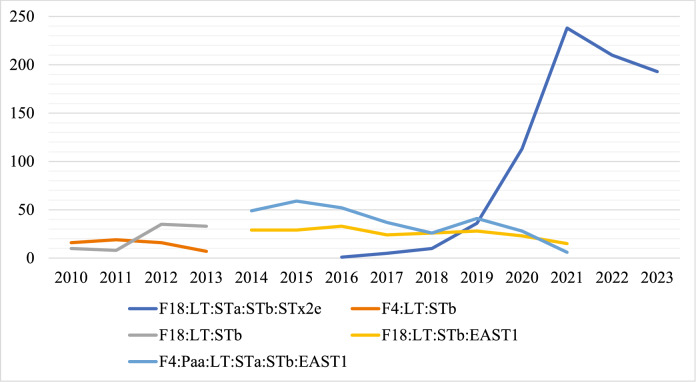
**Pathogenic** ***E. coli*** **virotypes detection over the study period at the ISU-VDL. Each color represents a different virotype**. The horizontal labels represent some of the most frequently detected virotypes and the most contemporary detected virotype.

The virotypes frequency of detection have changed over the years (Additional file 2), and the F18:LT:STa:STb:Stx2e virotype has increased in frequency since 2017 (Figure 3); Overall, it is the virotype most frequently detected (Table 3). The following most frequently detected virotypes were F4:Paa:LT:STa:STb:EAST1 (9.90%), F18:AIDA:Stx2e (0.57%), F5:STa (0.06%), AIDA:STa:STb:EAST1 (0.03%), respectively (Table 4), and (Additional files 3, 4, 5).

**Table 3:** **Number and frequency of F18 virulence factor combinations associated with the cases of PWC in the U.S. from 2010 to 2023**.

F18 Virulence factor combination	Number of isolates possessing combination (n)	Frequency of detection (%)
F18:LT:STa:STb:Stx2e	873	27.71%
F18:LT:STb:EAST1	215	6.83%
F18:LT:STa:STb:EAST1:Stx2e	141	4.48%
F18:LT:STa:STb:EAST1:Stx1:Stx2e	129	4.10%
F18:LT:STb:EAST1:Stx2e	114	3.62%
F18:LT:STb:EAST1:Stx1	101	3.21%
F18:LT:STb	90	2.86%
F18:LT:STa:STb:EAST1	55	1.75%
F18:LT:STa:STb	50	1.59%
F18:LT:STb:EAST1:Stx1:Stx2e	49	1.56%
F18:Paa:LT:STa:STb:Stx2e	34	1.08%
Others*	350	11.11%

^*^Other F18 virulence factor combinations (number and frequency of detection): F18:LT:STa:STb:EAST1:Stx1 (29–0.92%); F18:Paa:LT:STb:EAST1 (26–0.83%); F18:Paa:LT:STb:EAST1:Stx2e (26–0.83%); F18:STa:STb (24–0.76%); F18:Stx2e (24–0.76%); F18:LT:STb:Stx2e (22–0.70%); F18:Paa:EAST1 (21–0.67%); F18:STa:STb:Stx2e (19–0.60%); F18:LT:STa:Stx2e (14–0.44%); F18:Paa:EAST1:Stx1 (13–0.41%); F18:Paa:LT:STb:EAST1:Stx1 (12–0.38%); F18:LT:STa:STb:Stx2e (11–0.35%); F18:Paa:EAST1:Stx2e (11–0.35%); F18:Paa:LT:STb:EAST1:Stx1:Stx2e (7–0.22%); F18:LT (6–0.19%); F18:LT:Stx2e (5–0.16%); F18:EAST1:Stx2e (4–0.13%); F18:LT:STa:EAST1:Stx1:Stx2e (4–0.13%); F18:LT:STb:EAST1:Stx2e (4–0.13%); F18:Paa:EAST1:Stx1:Stx2e (4–0.13%); F18:EAST1 (3–0.10%); F18:LT:STa:EAST1:Stx2e (3–0.10%); F18:LT:STb:EAST1:Stx2 (3–0.10%); F18:STa:STb:Stx2e (3–0.10%); F18:STa:Stx2e (3–0.10%); F18:LT:STa:STb:EAST1:Stx1:Stx2 (2–0.06%); F18:LT:STa:STb:Stx2 (2–0.06%); F18:Paa (2–0.06%); F18:Paa:LT:STa:STb:EAST1:Stx1 (2–0.06%); F18:Paa:LT:STa:STb:EAST1:Stx1:Stx2e (2–0.06%); F18:Paa:LT:STa:STb:EAST1:Stx2e (2–0.06%); F18:Paa:STa:STb (2–0.06%); F18:STa (2–0.06%); F18:STa:STb:EAST1 (2–0.06%); F18:STa:STb:EAST1:Stx1:Stx2e (2–0.06%); F18:EAEA:LT:STa:STb:EAST1:Stx2e (1–0.03%); F18:EAEA:LT:STa:STb:Stx2e (1–0.03%); F18:EAEA:LT:STb:EAST1 (1–0.03%); F18:EAEA:LT:STb:EAST1:Stx1:Stx2e (1–0.03%); F18:EAEA:LT:STb:EAST1:Stx2 (1–0.03%); F18:EAEA:STa:STb:EAST1 (1–0.03%); F18:EAST1:Stx1 (1–0.03%); F18:LT:EAST1:Stx2e (1–0.03%); F18:LT:STa:STb:EAST1:Stx2 (1–0.03%); F18:LT:STa:STb:EAST1:Stx2e (1–0.03%); F18:LT:STa:Stx2e (1–0.03%); F18:LT:STb:EAST1:Stx1:Stx2 (1–0.03%); F18:LT:STb:Stx1 (1–0.03%); F18:LT:STb:Stx2e (1–0.03%); F18:LT:Stx2e (1–0.03%); F18:Paa:LT:STa:STb:EAST1 (1–0.03%); F18:Paa:LT:STa:STb:EAST1:Stx2e (1–0.03%); F18:Paa:LT:STb:Stx2e (1–0.03%); F18:Paa:STa:STb:EAST1 (1–0.03%); F18:Paa:Stx2e (1–0.03%); F18:STa:STb:Stx1 (1–0.03%); F18:STb (1–0.03%); F18:STb:EAST1 (1–0.03%); F18:Stx1 (1–0.03%); F18:Stx2 (1–0.03%); F18:Stx2e (1–0.03%).

**Table 4:** **Number and frequency of F4 (K88) virulence factor combinations associated with cases of PWC in the U.S. from 2010 to 2023**.

Virulence factor combination	Number of isolates possessing combination (n)	Frequency of detection (%)
F4:Paa:LT:STa:STb:EAST1	312	9.90%
F4:LT:STb:EAST1	107	3.40%
F4:LT:STb	71	2.25%
F4:Paa:LT:STa:STb:EAST1:Stx1	55	1.75%
F4:LT:STa:STb	49	1.56%
F4:Paa:LT:STb:EAST1	47	1.49%
F4:LT:STb:EAST1:Stx1	35	1.11%
Others*	149	4.73%

^*^Other virulence factor combinations (number and frequency of detection): F4:STa:STb (31–0.98%); F4:Paa:STa:STb:EAST1 (22–0.70%); F4:Paa:LT:STb:EAST1:Stx1 (15–0.48%); F4:Paa:STa:STb:EAST1:Stx1 (11–0.35%); F4:LT:STa:STb:EAST1 (10–0.32%); F4:LT (8–0.25%); F4:STa:STb:EAST1 (6–0.19%); F4:STa:STb:Stx1 (6–0.19%); F4:STa (4–0.13%); F4:STa:STb:Stx2e (4–0.13%); F4:LT:STa:STb:EAST1:Stx1 (3–0.10%); F4:Paa:STa:STb:Stx2e (3–0.10%); F4:EAST1:Stx1 (2–0.06%); F4:LT:STa:STb:Stx2 (2–0.06%); F4:Paa:LT:STa:STb:EAST1:Stx2e (2–0.06%); F4:STa (2–0.06%); F4:EAEA:Paa (1–0.03%); F4:EAST1 (1–0.03%); F4:LT (1–0.03%); F4:LT:STa:EAST1 (1–0.03%); F4:Paa:EAST1 (1–0.03%); F4:Paa:LT:STa:EAST1 (1–0.03%); F4:Paa:LT:STa:EAST1 (1–0.03%); F4:Paa:LT:STb (1–0.03%); F4:Paa:STa:STb (1–0.03%); F4:Paa:STb:EAST1 (1–0.03%); F4:STa:STb:EAST1:Stx1 (1–0.03%); F4:STa:STb:Stx1:Stx2 (1–0.03%); F4:STb:STb:EAST1 (1–0.03%); F4:STb:EAST1:Stx1 (1–0.03%); F4:STb:EAST1:Stx2 (1–0.03%); F4:Stx2 (1–0.03%); F4:Stx2e (1–0.03%); F4:LT:STa (1–0.03%); F4:STb (1–0.03%).

Smooth:mucoid colony morphology was associated with a greater likelihood of PWC (*P-*value > 0.001) compared to all other morphologies and greater than smooth, intermediate, and rough, respectively (Table 5).

**Table 5:** **Comparison of morphologies and the likelihood of PWC in pigs**.

Morphologies		Risk estimates	95% CI	*P*-value
Smooth mucoid	Rough	0.993	0.987–0.998	< 0.001
Smooth mucoid	Intermediate	0.968	0.956–0.979	
Smooth mucoid	Smooth	0.758	0.733–0.782	
Rough	Smooth	0.021	0.004–0.037	
Rough	Smooth mucoid	0.006	0.001–0.012	
Smooth mucoid	All others	0.282	0.266–0.298	

A spatial and temporal analysis of the most frequently detected virotype in this study demonstrated a crescent distribution across states over the years of the study (Additional file 6). The cases of PWC positive for isolates of this virotype were more densely located in the northwest and east of Iowa and northeast of Indiana in 2021 (Additional file 7).

## Discussion

This study described the rate of PWC and the frequency of fimbrial and toxin gene detection in pathogenic *E. coli* from pigs with PWD across different U.S. states. PWC rate significantly increased in 2012, 2013, 2019, 2020, and 2021, respectively, compared with the previous year, and the F18 and F4 adhesin genes were most often detected (69.87% and 26.19%, respectively). Factors associated with the rate increase in number of cases and changes in fimbrial type frequency of detection were not evaluated; however, interestingly, the relative frequency of isolates positive for F4 or F18 fimbrial types in this study differs from that previously observed in pigs with diarrhea in the U.S. (64.6% for F4 and 34.3% for F18, respectively) [6]. A higher prevalence of F4 (45.1%) compared with F18 (33.9%) has been reported in some countries in Europe [21]; nevertheless, similar to this study, data from Slovakia and Poland have shown a higher prevalence of F18 fimbriae [22, 38].

A lower frequency of isolates with F5 (0.25%), F41 (0.13%), AIDA (0.10%), and multiple adhesins (3.46%) associated with PWC were found in this study. F5 and F41 are usually associated with neonatal and pre-weaning diarrhea [25]; however, the detection of these fimbriae from post-weaning pigs has been described [1, 19, 20, 22]. Regarding toxins and non-fimbrial adhesins, STb was the most prevalent detected (93.33%), followed by LT (89.49%), STa (63.30%), EAST1 (53.71%), Stx2e (50.19%), Stx2 (48.51%), Paa (21.40%), Stx1 (16.67%), and EAEA (0.35%), respectively. These toxins and adhesins were detected simultaneously with different fimbriae types, characterizing different virulence factor combinations more recently classified as virotypes [7].

The virotype most frequently detected was F18:LT:STa:STb:Stx2e (27.71%), followed by F4:Paa:LT:STa:STb:EAST1 (9.90%), F18:AIDA:Stx2e (0.57%), F5:STa (0.06%), and AIDA:STa:STb:EAST1 (0.03%). The genes LT, STa, and STb are usually associated with diarrhea and the ETEC pathotype, whereas Stx2e is associated with ED and the STEC pathotype [2]. Thus, pathotypes containing genes usually assigned to both ETEC and STEC are classified as hybrid [7, 14]. Isolates belonging to the hybrid ETEC and STEC pathotype have been described as highly virulent in diarrheic pigs in Europe [8–10] and China [15] and are frequently reported in cases of PWD and ED in piglets [11–14]. This study reports the prevalence of a hybrid pathotype associated with PWC across different states in the U.S.

The smooth:mucoid colony morphology was likely associated with PWC (Table 9). Although limited literature information and limitations of laboratory data, the authors believe that it is of value to describe the type of morphology associated with confirmed PWC. Morphology is used as a first macroscopic screening and the first indication of potentially pathogenic *E. coli* in the laboratory; although the presence of the hemolysin is not considered to be a virulence factor for ETEC and STEC, hemolysis is frequently used as a marker for pathogenicity in F4 and F18 isolates [25]. Although the morphology associated with ETEC and STEC pathotypes and PWC may vary, the authors speculate that smooth:mucoid morphology in confirmed PWC cases may be a potential marker for pathogenicity.

The temporal analysis of cases associated with the F18:LT:STa:STb:Stx2e virotype demonstrated crescent distribution across U.S. states from 2016 to 2023. The greater intensity of the cases in Iowa is likely associated with its higher number of positive cases in this area, as shown in Table 1; interestingly, a higher intensity was also observed in Indiana in 2021. This study did not evaluate factors associated with the increase in the virotype distribution. However, this certainly has implications for the swine industry due to the potential of this hybrid pathotype to cause more severe disease, as already discussed in this section.

The underlying reasons for the higher frequency of detection of F18:LT:STa:STb:Stx2e virotype needs further investigation. Non-susceptibility to antimicrobials, high density of swine farms, direct and indirect contacts between farms through pig movements and people (e.g., veterinarians, transporters), and possibly antimicrobial prescriptions habit in different regions are described as potential factors that could facilitate emergence or dissemination of isolates [39]; however, these factors were not investigated in this study. In addition, the authors understand the importance of full characterization of the isolate, including the presence of antimicrobial-resistant genes, to help understand potential mechanisms associated with its higher detection frequency.

Similar to previously reported studies based on diagnostic data [31, 40, 41], interpreting findings using large datasets created based on specific information from diagnostic cases from a single laboratory deserves caution. Disease detection and diagnosis results dependent on what was submitted, what was targeted by the submitter, and what was defined as relevant by the group of diagnosticians may fall under the selection and cognitive bias, respectively [40]. Yet, veterinary diagnostic data has great potential to support local, regional, and national animal health, provide near real-time situational awareness, and support outbreak response [42, 43]. This study describes the diversity in the frequency of detection of pathogenic *E. coli* associated with PWC in the U.S. and a hybrid contemporary virotype.

The virulence factors and morphology of enterotoxigenic *E. coli* associated with PWC across different states in the U.S. from 2010 to 2023 were diverse; nevertheless, the virotype F18:LT:STa:STb:Stx2e predominated and increased in frequency during this time period. Confirming the presence of a hybrid and virulent pathotype associated with clinical cases of PWD has practical relevance for the swine industry regarding preventing and controlling PWC. This information will help swine veterinarians, producers, and stakeholders better understand the PWC industry scenario and its implications for PWD outbreaks.

## Supplementary Information

**Additional file 1: Number of pathogenic** ***E. coli***^***1***^** isolates per year from confirmed PWC cases in different U.S. states**.

**Additional file 2: Pathogenic *E. coli* virotypes most frequently detected over the study period**.

**Additional file 3: Number and frequency of multiple fimbrial types and virulence factor combinations associated with the cases of PWC in the U.S. from 2010 to 2023**.

**Additional file 4: Number and frequency of F5virulence factor combinations associated with cases of PWC in the U.S. from 2010 to 2023**.

**Additional file 5: Number and frequency of AIDA virulence factor combinations associated with cases of PWC across the U.S. from 2010 to 2023**.

**Additional file 6: Distribution of cases of PWC associated with *E. coli* isolates possessing F18:LT:STa:STb:Stx2e virulence factor combination across U.S. states from 2016 to 2023**.

**Additional file 7: Heatmap based on Kernel density demonstrating the distribution of cases of PWC associated with** ***E. coli* isolates possessing F18:LT:STa:STb:Stx2e virulence factor combination across U.S. Midwestern states from 2020 through 2023.**

## Data Availability

The datasets presented in this article are not readily available due to client confidentiality, but the data can be searched for analysis by authorized personnel only. Specific requests to access the datasets should be directed to Marcelo Almeida, malmeida@iastate.edu.
