# Supplementary material for: Description of a contemporary pathogenic Escherichia coli isolated from pigs with post-weaning diarrhea in the United States from 2010 to 2023
Source: Vet Res. 2025 Jul 1;56:130. doi: 10.1186/s13567-025-01568-y (PMC12218006; doi:10.1186/s13567-025-01568-y)
Supplement: Supplementary file 1 — Additional file 1: Number of pathogenic E. coli1 isolates per year from confirmed PWC cases in different U.S. states. [file 13567_2025_1568_MOESM1_ESM.docx]

**Additional file 1** **Number of pathogenic *E. coli*^1^ isolates per year from confirmed PWC cases in different U.S. states.**

| States | 2010 | 2011 | 2012 | 2013 | 2014 | 2015 | 2016 | 2017 | 2018 | 2019 | 2020 | 2021 | 2022 | 2023 | Total |
| --- | --- | --- | --- | --- | --- | --- | --- | --- | --- | --- | --- | --- | --- | --- | --- |
| IA |  |  |  |  | 39 | 57 | 81 | 90 | 90 | 133 | 177 | 366 | 221 | 144 | 1398 |
| IL |  |  |  |  | 6 | 15 | 9 | 5 | 13 | 17 | 23 | 41 | 30 | 21 | 180 |
| MO |  |  |  |  | 3 | 5 | 9 | 9 | 4 | 12 | 25 | 46 | 32 | 32 | 177 |
| NC |  |  |  |  | 8 | 3 | 20 | 16 | 17 | 13 | 11 | 20 | 22 | 36 | 166 |
| MN |  |  |  |  | 3 | 8 | 6 | 7 | 9 | 13 | 13 | 33 | 31 | 20 | 143 |
| IN |  |  |  |  | 7 | 4 | 5 | 1 | 8 | 8 | 16 | 28 | 34 | 21 | 132 |
| OH |  |  |  |  |  | 3 | 7 | 3 | 3 | 13 | 25 | 22 | 20 | 14 | 110 |
| NE |  |  |  |  | 2 | 4 | 6 | 4 | 9 | 7 | 9 | 5 | 5 | 2 | 53 |
| SD |  |  |  |  | 3 | 2 | 3 | 4 | 4 | 7 | 5 | 10 | 3 | 8 | 49 |
| PA |  |  |  |  |  | 1 | 3 | 1 | 1 | 4 | 11 | 14 | 5 | 7 | 47 |
| MI |  |  |  |  |  | 1 | 1 |  | 2 | 5 |  | 1 | 6 | 6 | 22 |
| UT |  |  |  |  |  |  |  |  | 1 | 1 | 3 | 7 |  | 1 | 13 |
| KS |  |  |  |  | 1 |  |  |  |  |  | 1 | 1 | 3 | 3 | 9 |
| OK |  |  |  |  |  | 2 |  |  | 1 | 2 | 1 | 1 |  | 1 | 8 |
| WI |  |  |  |  |  |  |  | 1 | 1 |  |  |  | 1 | 3 | 6 |
| VA |  |  |  |  | 1 | 1 | 1 | 1 |  |  |  |  |  |  | 4 |
| CO |  |  |  |  |  |  |  |  |  |  | 1 | 1 | 1 |  | 3 |
| ID |  |  |  |  |  |  |  | 1 | 1 |  |  |  |  | 1 | 3 |
| AR |  |  |  |  | 1 |  |  |  |  | 1 |  |  |  |  | 2 |
| SC |  |  |  |  |  |  |  |  |  | 1 |  | 1 |  |  | 2 |
| TX |  |  |  |  |  |  | 1 |  |  |  |  |  |  | 1 | 2 |
| AK |  |  |  |  |  |  |  |  |  |  |  |  | 1 |  | 1 |
| AZ |  |  |  |  |  |  |  |  |  |  |  |  |  | 1 | 1 |
| CA |  |  |  |  |  |  |  |  | 1 |  |  |  |  |  | 1 |
| KY |  |  |  |  |  |  |  |  |  |  |  |  |  | 1 | 1 |
| MT |  |  |  |  |  |  |  |  | 1 |  |  |  |  |  | 1 |
| NY |  |  |  |  |  |  |  |  |  |  |  |  |  | 1 | 1 |
| TN |  |  |  |  |  |  |  |  |  |  |  |  |  | 1 | 1 |
| WA |  |  |  |  |  |  |  |  |  |  | 1 |  |  |  | 1 |
| NA* | 49 | 65 | 95 | 128 | 120 | 48 | 23 | 23 | 11 | 15 | 11 | 11 | 5 | 9 | 613 |
| Total | 49 | 65 | 95 | 128 | 194 | 154 | 175 | 166 | 177 | 252 | 333 | 608 | 420 | 334 | 3150 |

^1^ Isolates positive for one or more tested virulence genes. ^*^Isolates from cases with no US states informed.
