# Supplementary material for: Description of a contemporary pathogenic Escherichia coli isolated from pigs with post-weaning diarrhea in the United States from 2010 to 2023
Source: Vet Res. 2025 Jul 1;56:130. doi: 10.1186/s13567-025-01568-y (PMC12218006; doi:10.1186/s13567-025-01568-y)
Supplement: Supplementary file 2 — Additional file 2: Pathogenic E. coli virotypes most frequently detected over the study period. [file 13567_2025_1568_MOESM2_ESM.docx]

F18:LT:STb:EAST1

F18:LT:STa:STb:STx2e

F18:LT:STb

F4:LT:STb

F4:Paa:LT:STa:STb:EAST1
