# Supplementary material for: Description of a contemporary pathogenic Escherichia coli isolated from pigs with post-weaning diarrhea in the United States from 2010 to 2023
Source: Vet Res. 2025 Jul 1;56:130. doi: 10.1186/s13567-025-01568-y (PMC12218006; doi:10.1186/s13567-025-01568-y)
Supplement: Supplementary file 3 — Additional file 3: Number and frequency of multiple fimbrial types and virulence factor combinations associated with the cases of PWC in the U.S. from 2010 to 2023. [file 13567_2025_1568_MOESM3_ESM.docx]

**Additional file 3** **Number and frequency of multiple fimbrial types and virulence factor combinations associated with the cases of PWC in the U.S. from 2010 to 2023.**

| Virulence factor combination | Number of isolates possessing combination (n) | Frequency of detection (%) |
| --- | --- | --- |
| F18:AIDA:Stx2e | 18 | 0.57% |
| F18:AIDA | 6 | 0.19% |
| F4:AIDA:Paa:LT:STa:STb:EAST1:STx1 | 3 | 0.10% |
| F18:AIDA:EAST1:Stx1:Stx2:Stx2e | 3 | 0.10% |
| F18:AIDA:LT:STb:EAST1:Stx1 | 3 | 0.10% |
| F18:AIDA:STa:STb:Stx2:Stx2e | 3 | 0.10% |
| F18:F4:LT:STb:EAST1:Stx1 | 3 | 0.10% |
| F18:K99:LT:STb:Stx2 | 3 | 0.10% |
| F4:AIDA:Paa:STb:EAST1 | 2 | 0.06% |
| F41:F5:STa | 2 | 0.06% |
| F18:AIDA:EAST1:Stx2:Stx2e | 2 | 0.06% |
| F18:AIDA:LT:STa:STb:EAST1:Stx1:Stx2e | 2 | 0.06% |
| F18:AIDA:LT:STb:EAST1 | 2 | 0.06% |
| F18:F4:AIDA:Stx2 | 2 | 0.06% |
| F18:F4:LT:STa:STb | 2 | 0.06% |
| F18:F4:LT:STb:Stx2e | 2 | 0.06% |
| F18:F4:Paa:LT:STa:STb:EAST1 | 2 | 0.06% |
| F18:F4:Paa:LT:STa:STb:EAST1:Stx1 | 2 | 0.06% |
| F18:F4:Paa:STa:STb:EAST1 | 2 | 0.06% |
| F18:F4:Paa:STa:STb:EAST1:Stx2 | 2 | 0.06% |
| F18:F4:Paa:STa:STb:Stx2 | 2 | 0.06% |
| Others* | 41 | 1.30% |

*Other virulence factor combinations (1-0.03% number and frequency of detection, respectively). F4:AIDA:Paa:LT:STb:EAST1 F4:AIDA:STa:STb:EAST1; F4:AIDA:STb:EAST1:Stx1; F4:F41:AIDA:Paa:LT:STa:STb:EAST1:Stx1; F41:AIDA:STb:Stx1:Stx2e; F41:F5:Paa:STa; F41:F5:Paa:STa:EAST1:Stx1; F5:EAEA:STa:EAST1:Stx1; F18:987P:STb; F18:AIDA:EAEA:Paa; F18:AIDA:EAEA:Paa:LT:STb:EAST1; F18:AIDA:LT:STb:EAST1:Stx2e; F18:AIDA:Paa:LT:STa:STb:EAST1:Stx1:Stx2e; F18:AIDA:Paa:LT:STb:EAST1:Stx1; F18:AIDA:Paa:LT:STb:EAST1:Stx2e; F18:AIDA:Paa:Stx1:Stx2e; F18:AIDA:STa:STb; F18:AIDA:STa:STb:EAST1:Stx2e; F18:AIDA:STb:Stx2e; F18:AIDA:Stx1; F18:AIDA:Stx1:Stx2e; F18:AIDA:Stx2e; F18:F4:AIDA:Paa:LT:STa:STb:EAST1:Stx2e; F18:F4:F41:EAST1:Stx1; F18:F4:F5:LT:STa:STb:Stx2e; F18:F4:LT; F18:F4:LT:STa:STb:Stx2e; F18:F4:Paa:EAST1:Stx1:Stx2e; F18:F4:Paa:LT:STa:STb:EAST1:Stx2; F18:F4:Paa:LT:STa:STb:EAST1:Stx2e; F18:F4:Paa:LT:STb:EAST1:Stx1; F18:F4:Paa:STa:STb; F18:F4:STb:EAST1; F18:F41:LT; F18:F41:LT:STb; F18:F5:AIDA:Stx1:Stx2; F18:F5:LT:STa:STb:EAST1:Stx2e; F18:F5:LT:STb:EAST1:Stx1:Stx2; F18:F4:Paa; F18:F4:Paa:STb; F18:F4:Stx2.
