# Supplementary material for: Description of a contemporary pathogenic Escherichia coli isolated from pigs with post-weaning diarrhea in the United States from 2010 to 2023
Source: Vet Res. 2025 Jul 1;56:130. doi: 10.1186/s13567-025-01568-y (PMC12218006; doi:10.1186/s13567-025-01568-y)
Supplement: Supplementary file 4 — Additional file 4: Number and frequency of F5virulence factor combinations associated with cases of PWC in the U.S. from 2010 to 2023. [file 13567_2025_1568_MOESM4_ESM.docx]

**Additional file 4** **Number and frequency of F5 (K99) virulence factor combinations associated with cases of PWC in the U.S. from 2010 to 2023.**

| Virulence factor combination | | | | Number of isolates possessing combination(n) | Frequency of detection (%) |
| --- | --- | --- | --- | --- | --- |
| F5:STa |  |  |  | 2 | 0.06% |
| F5:STa:STb | |  |  | 2 | 0.06% |
| F5:Paa:LT:STa:STb:EAST1:Stx1 | | | | 1 | 0.03% |
| F5:Paa:LT:STb:EAST1 | | |  | 1 | 0.03% |
| F5:Paa:STb:EAST1 | |  |  | 1 | 0.03% |
| F5:STa:Stx2e | |  |  | 1 | 0.03% |
