# Supplementary material for: Description of a contemporary pathogenic Escherichia coli isolated from pigs with post-weaning diarrhea in the United States from 2010 to 2023
Source: Vet Res. 2025 Jul 1;56:130. doi: 10.1186/s13567-025-01568-y (PMC12218006; doi:10.1186/s13567-025-01568-y)
Supplement: Supplementary file 5 — Additional file 5: Number and frequency of AIDA virulence factor combinations associated with cases of PWC across the U.S. from 2010 to 2023. [file 13567_2025_1568_MOESM5_ESM.docx]

**Additional file 5** **Number and frequency of AIDA virulence factor combinations associated with cases of PWC across the U.S. from 2010 to 2023.**

| Virulence factor combination | Number of isolates possessing combination (n) | Frequency of detection (%) |
| --- | --- | --- |
| AIDA:STa:STb:EAST1 | 1 | 0.03% |
| AIDA:EAEA:Paa | 1 | 0.03% |
| AIDA:STb:EAST1:Stx1 | 1 | 0.03% |
