# Supplementary figures and images for: Description of a contemporary pathogenic Escherichia coli isolated from pigs with post-weaning diarrhea in the United States from 2010 to 2023
Source: Vet Res. 2025 Jul 1;56:130. doi: 10.1186/s13567-025-01568-y (PMC12218006; doi:10.1186/s13567-025-01568-y)

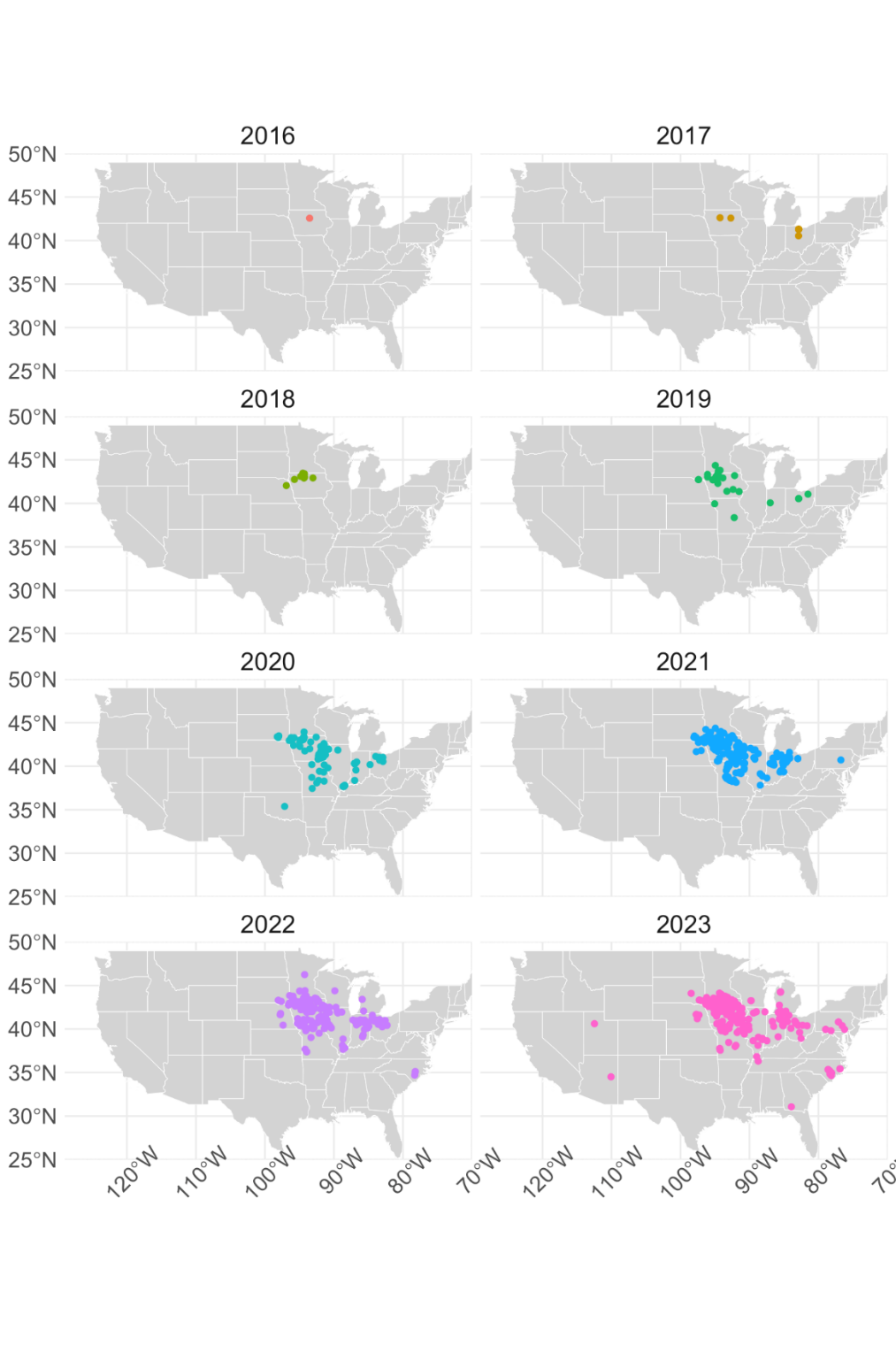

Supplement: Supplementary file 6 — Additional file 6: Distribution of cases of PWC associated with E. coli isolates possessing F18:LT:STa:STb:Stx2e virulence factor combination across U.S. states from 2016 to 2023. [file 13567_2025_1568_MOESM6_ESM.docx]

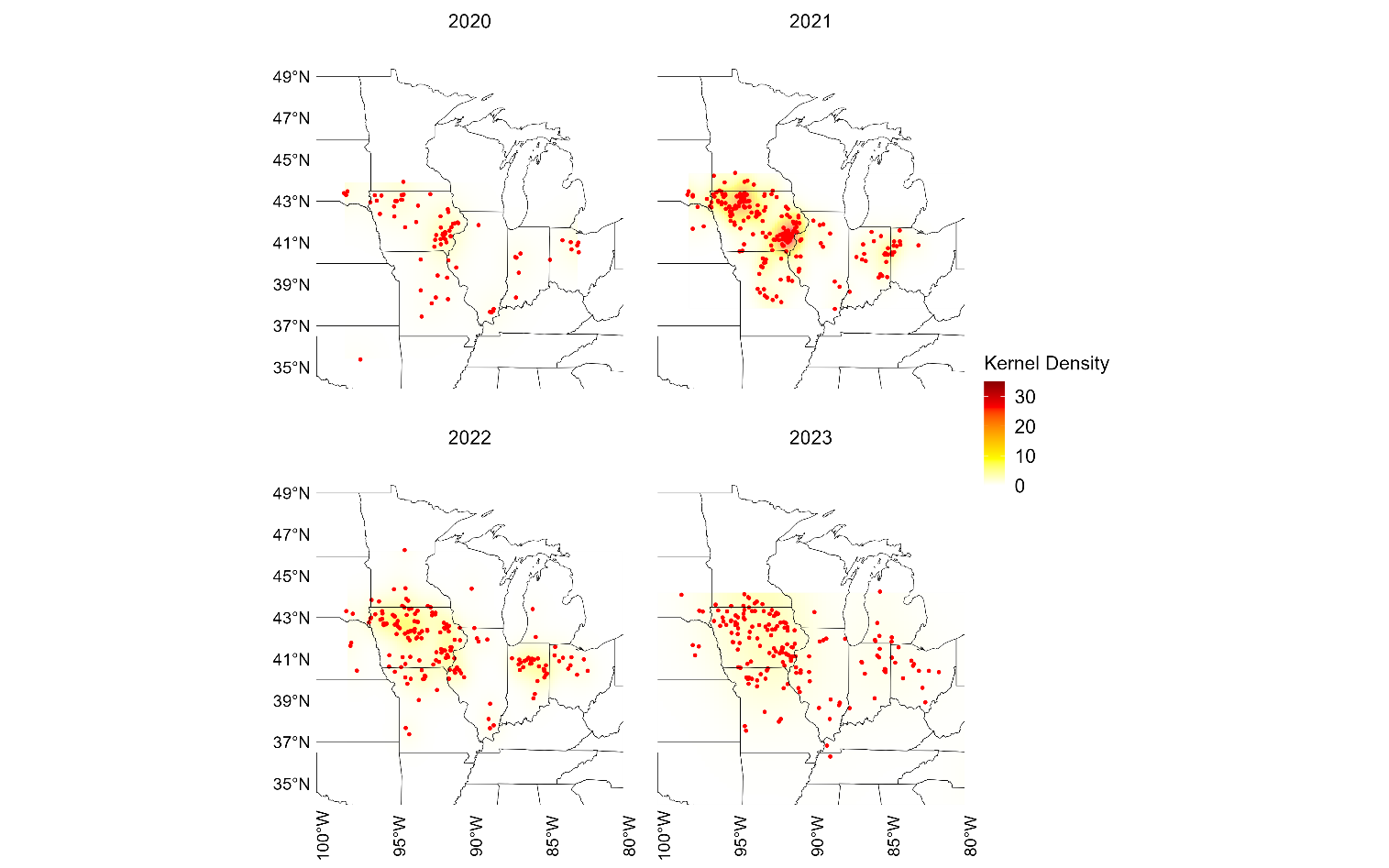

Supplement: Supplementary file 7 — Additional file 7: Heatmap based on Kernel density demonstrating the distribution of cases of PWC associated with E. coli isolates possessing F18:LT:STa:STb:Stx2e virulence factor combination across U.S. Midwestern states from 2020 through 2023. [file 13567_2025_1568_MOESM7_ESM.docx]
